# IMPACT OF FRAILTY SCREENING IN A BURN CENTER: A QUALITY IMPROVEMENT PROJECT

**DOI:** 10.1093/geroni/igae098.0718

**Published:** 2024-12-31

**Authors:** Purvi Patel

**Affiliations:** University of Chicago Medicine, Chicago, Illinois, United States

## Abstract

Burn injuries create significant risk for the development of new or worsening frailty. Identifying risk for frailty is key to recognizing patients who are at risk for negative outcomes who may benefit from a comprehensive geriatric assessment (CGA). A quality improvement project was conducted in a burn center setting with the primary aim to determine if implementation of the FRAIL scale screening tool impacted frequency of consultation to the geriatric medicine team for a CGA within a 10-week pilot period. Secondary aims were to evaluate feasibility of implementation and to stratify patients’ risk of frailty into categories based on the tool measurements: robust, pre-frail, or frail. The project included 44 patients (22 in the pre-frailty tool group and 22 in the post-frailty tool group). The data revealed a three-fold increase in the number of consultations to the geriatric medicine team between the pre (n=5) and post (n=15) groups with statistical significance (p =.002). The frailty tool was most often administered on the first (54.5%) or second (31.8%) hospital day. In the post group, stratification of frailty risk was 27.3% robust, 45.4% pre-frail, and 27.3% frail. The FRAIL scale is shown to be useful in identifying patients who would benefit from a CGA compared to routine clinical practice. The tool is feasible to implement in a burn center. The clinical significance of the majority of patients being classified as pre-frail and frail underscores the need to identify patients’ frailty risk and implement measure to mitigate adverse outcomes.

